# The Pyridoxal 5′-Phosphate (PLP)-Dependent Enzyme Serine Palmitoyltransferase (SPT): Effects of the Small Subunits and Insights from Bacterial Mimics of Human hLCB2a HSAN1 Mutations

**DOI:** 10.1155/2013/194371

**Published:** 2013-09-23

**Authors:** Ashley E. Beattie, Sita D. Gupta, Lenka Frankova, Agne Kazlauskaite, Jeffrey M. Harmon, Teresa M. Dunn, Dominic J. Campopiano

**Affiliations:** ^1^EastChem School of Chemistry, The University of Edinburgh, West Mains Road, Edinburgh EH9 3JJ, UK; ^2^Department of Biochemistry and Molecular Biology, Uniformed Services University of the Health Sciences, Bethesda, MD 20184-4779, USA; ^3^The Edinburgh Cell Wall Group, Institute of Molecular Plant Sciences, School of Biological Sciences, The University of Edinburgh, Daniel Rutherford Building, Edinburgh EH9 3JH, UK; ^4^Department of Pharmacology, Uniformed Services University of the Health Sciences, Bethesda, MD 20184-4779, USA

## Abstract

The pyridoxal 5′-phosphate (PLP)-dependent enzyme serine palmitoyltransferase (SPT) catalyses the first step of *de novo* sphingolipid biosynthesis. The core human enzyme is a membrane-bound heterodimer composed of two subunits (hLCB1 and hLCB2a/b), and mutations in both hLCB1 (e.g., C133W and C133Y) and hLCB2a (e.g., V359M, G382V, and I504F) have been identified in patients with hereditary sensory and autonomic neuropathy type I (HSAN1), an inherited disorder that affects sensory and autonomic neurons. These mutations result in substrate promiscuity, leading to formation of neurotoxic deoxysphingolipids found in affected individuals. Here we measure the activities of the hLCB2a mutants in the presence of ssSPTa and ssSPTb and find that all decrease enzyme activity. High resolution structural data of the homodimeric SPT enzyme from the bacterium *Sphingomonas paucimobilis* (*Sp* SPT) provides a model to understand the impact of the hLCB2a mutations on the mechanism of SPT. The three human hLCB2a HSAN1 mutations map onto *Sp* SPT (V246M, G268V, and G385F), and these mutant mimics reveal that the amino acid changes have varying impacts; they perturb the PLP cofactor binding, reduce the affinity for both substrates, decrease the enzyme activity, and, in the most severe case, cause the protein to be expressed in an insoluble form.

## 1. Introduction

**Figure 1:**
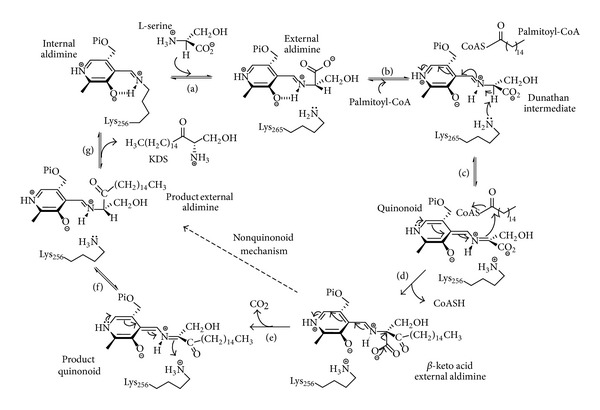
Proposed SPT mechanism. (a) Transamination of internal aldimine by incoming L-serine to form PLP-L-serine external aldimine intermediate; (b) binding of palmitoyl-CoA causes conformational change and deprotonation of external aldimine by Lys265 to form quinonoid/carbanion intermediate; (c) quinonoid/carbanion attack of the thioester; (d) formation of *β*-keto acid and CoASH release; (e) release of CO_2_ to form KDS product quinonoid; (f) reprotonation of KDS; (g) transamination to release KDS and restore the internal aldimine with PLP-bound to Lys265.

Sphingolipids (SLs) are membrane components found in all eukaryotes, some prokaryotes, and viruses. SLs and their downstream metabolites (such as ceramide) play important roles in mediating cell-stress response and cell proliferation and in regulating the cell cycle and apoptosis [1–3]. Deficiencies in SL metabolism have been implicated in several diseases that include cancer and diabetes, as well as neurodegenerative disorders such as Alzheimer's [4, 5]. The first enzyme of the *de novo *sphingolipid biosynthetic pathway present in all SL-producing organisms is the pyridoxal 5′-phosphate- (PLP-) dependent serine palmitoyltransferase ((SPT) EC 2.3.1.50) [6]. It catalyses the condensation of L-serine with palmitoyl coenzyme-A to generate 3-ketodehydrosphinganine (KDS) that forms the sphingoid base backbone of all SLs. The 3D structure of the soluble, homodimeric SPT from the SL-producing organism *S. paucimobilis* (*Sp* SPT) was published by our group in 2007. It is a member of the PLP superfamily (fold type I) and contains three domains: N-terminal, central, and C-terminal [7]. Based on extensive structural and biochemical analyses of this enzyme, a working SPT mechanism involves the formation of a number of key intermediates (Figure 1) [7]. An initial PLP-bound internal aldimine (covalently attached to Lys265) is displaced by L-serine to generate a PLP:L-serine external aldimine. Binding of the C16 acyl-CoA thioester allows formation of a carbanion/quinonoid nucleophile by deprotonation of the external aldimine. After Claisen-like condensation, a *β*-keto acid is formed which subsequently loses CO_2_ to generate a PLP:KDS product aldimine which is then released from the enzyme, and the SPT returns to its PLP-bound internal aldimine form. We and others have investigated the catalytic roles of conserved active site SPT residues in this mechanism [8–11].

Eukaryotic SPT is a membrane-bound enzyme which contains two core subunits, LCB1 and LCB2 in yeast [12–14] and SPTLC1 and SPTLC2/3 in mammals [15–17]. For clarity we have adopted the nomenclature hLCB1, hLCB2a/b for the human subunits [18]. The hLCB1 and hLCB2 subunits display relatively high sequence homology to each other, but a comparison with other PLP-binding enzymes revealed that only the hLCB2a contains key catalytic residues including the lysine that binds PLP [19, 20]. In contrast, hLCB1 lacks these residues but contains other residues predicted to be involved in catalysis leading to the working hypothesis that the hLCB1/hLCB2a heterodimer has a single active site. Hornemann and colleagues suggested that the eukaryotic SPT may in fact be a higher order complex [21], and indeed recent results lend weight to that hypothesis. Also, the same group recently identified a second hLCB2 isoform, hLCB2b, also known as SPTLC3 with 68% sequence identity to hLCB2a that is predominantly expressed in the placenta and prefers shorter chain acyl-CoAs (C12 and C14) to generate short-chain SLs [22]. We identified two highly-related isoforms of a third “small subunit” in humans (ssSPTa and ssSPTb) which were shown to be crucial for maximal enzyme activity [18]. These are functionally orthologous to a previously-characterised small subunit (Tsc3p) discovered in yeast [23]. These ssSPTs can increase the catalytic activity of the hLCB1/hLCB2 complex up to 100 fold, and also influence the chain-length specificity of the acyl-CoA substrate, and, thus, impact directly on the concentrations and chemical nature of the SL pool. This complexity was further increased by the recent discovery of other components of a so-called SPOTS complex in yeast that is composed of LCB1/LCB2/Tsc3p, an ORM protein(s), and a phosphoinositide phosphatase, Sac1 [24, 25]. These ORM and Sac1 components are thought to play a role in modulating SPT activity and flux through the SL pathway by a phosphorylation-dependent mechanism [25–28].

**Figure 2:**
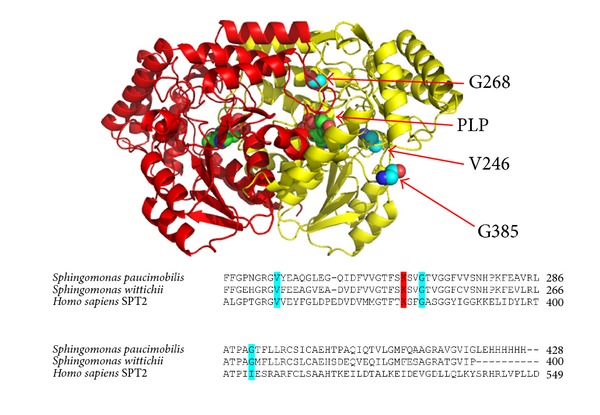
High resolution structure of *Sp *SPT homodimer PLP-L-serine external aldimine showing the position of the three hLCB2a HSAN1 related mutations (PDB:2W8J). Each monomer is coloured (red and yellow). Sequence alignment of bacterial *Sp* SPT (Uniprot code: Q93UV0) with bacterial *Sw *SPT (Uniprot code: A5VD79) and human hLCB2a (Uniprot code: O15270). The key catalytic lysine residue (K265) involved in the formation of the internal aldimine is coloured red, and the HSAN1 mutant mimics (V246M, G268V, and G385F) are coloured cyan.

Since SLs and ceramides are essential elements of cellular membranes that also play important roles in cell signalling, homozygous LCB1/LCB2 mutations that cause complete loss of SPT activity would be assumed to be lethal. However, there are very rare gain-of-function SPT mutations that have been identified and their genetic lineage has been studied [29–33]. These SPT mutations lead to hereditary sensory neuropathies (HSAN1) whose clinical outcomes include progressive distal sensory loss and severe ulcerations of the limbs. What causes the neuronal breakdown has been the subject of interesting debate. As well having an impact on the basal SPT activity, a new hypothesis suggests that the SPT mutations associated with HSAN1 lead to an enzyme lacking exquisite substrate specificity for L-serine. Penno and colleagues discovered that tissue from HSAN1 patients contains high levels of L-alanine and glycine-derived deoxy-SLs that have been shown to be toxic to HEK293 cells [32]. Four missense mutations in the hLCB1 subunit associated with HSAN1 (C133W, C133Y, V144D, and G387A) were among the first to be identified (Table 1) and have shown to have a negative effect on the SPT enzyme and sphingolipid production [29, 30, 34, 35]. Another mutation at residue C133 (C133R) was identified in a patient with a mild phenotype, but its impact on SPT activity has not been characterized [36]. The multisubunit, membrane-bound nature of the eukaryotic SPT has prevented a detailed structural and mechanistic analysis of the impact of the HSAN1 causing mutations on the enzyme. To gain insight into and explore the molecular basis of the HSAN1 genotype we have used the *Sp* SPT isoform as a model system. Both cysteine mutations were previously engineered into the *Sp* SPT (as N100W and N100Y) in our group [10]. These mutations result in a compromised SPT enzyme, severely impacting PLP binding, reducing affinity for the L-serine substrate, and causing catastrophic effects on catalytic activity. More recently, two more hLCB1 mutations (S331F and A352V) were identified. The S331F mutation resulted in symptoms far more severe than those associated with the more common HSAN1 mutations. Furthermore, a second mutation at amino acid position Ser331 (S331Y) is linked to a distinct syndrome phenotype [37, 38]. Interestingly, for the first time, three different mutations (V359M, G382V, and I504F) were recently identified in the hLCB2a subunit by Rotthier et al. [39]. They found that each mutation lowered the activity of the hLCB1/hLCB2a heterodimer and was also able to generate varying levels of deoxy-SLs. This group also modelled the three hLCB2a mutations onto the bacterial *Sp *SPT (V246M, G268V, and G385F) paving the way for the biochemical analysis that we describe here (Figure 2). In this report, we have studied the influence of the human small subunits on the activity of the HSAN1 hLCB2a mutations. As well as this, we have also characterised the bacterial mutant mimics using enzyme kinetics, spectroscopy, and molecular modelling to provide insight into the impact they have on PLP cofactor binding, catalytic activity, and/or substrate binding.

## 2. Materials and Methods

### 2.1. Chemicals and Molecular Biology Materials

Plasmids and competent cells were purchased from Novagen. All buffers and reagents were from Sigma. Palmitoyl-CoA was purchased from Avanti Lipids.

### 2.2. Methods

#### 2.2.1. Analysis of Human SPT Wild Type and HSAN1 Mutant Activity in the Presence of the ssSPTa and ssSPTb Subunits

The construction of yeast mutant strains lacking *lcb1*, *lcb2,* and *tsc3* was described previously [18, 31]. Yeast microsomes were prepared, and expression of recombinant human forms of each subunit, hLCB1, hLCB2a, ssSPTa, or ssSPTb, was visualised by immunoblotting using antibodies directed against hLCB1, hLCB2a, and the HA epitope as previously described [18]. Yeast microsomal SPT activity was assayed by measuring [^3^H] serine incorporation into long chain bases as previously described [18].

#### 2.2.2. Bacterial SPT Gene Cloning and Mutagenesis

The plasmid (pET 28a/*Sp* SPT) that we use to express *S. paucimobilis *SPT in *E. coli* was available at the start of this study and contains a six-histidine tag at the C-terminus [10]. This was used as a template, and all HSAN1 mutations were made using the Liu and Naismith [43] mutagenesis protocol with the following primers:

 5′CGGC***ATG***TACGAGGCGCAAG 3′ (V246M forward)
 5′TCGTA***CAT***GCCGCGCCCGTTG 3′ (V246M reverse)
  
 5′GGTC***GTC***ACAGTCGGCGGCTTC 3′ (G268V forward)
 5′ACTGT***GAC***GACCGATTTGGAG 3′ (G268V reverse)
  
 5′CGGCC***TTC***ACCTTCCTGCTG 3′ (G385F forward)
 5′AAGGTG***AAG***GCCGGGGTC 3′ (G385F reverse).

The bases mutated are shown in bold and italic. The isolated mutants were verified by DNA sequencing and mass spectrometry analysis of the purified enzyme.

#### 2.2.3. Cloning and Expression of *Sphingomonas paucimobilis* SPT (*Sp* SPT) and HSAN1 Mutant Mimics

The *Sp *SPT enzyme was prepared as previously described [10]. A single colony pET28a *Sp *SPT was grown overnight at 37°C in LB selected with kanamycin (30 *μ*g/mL) using *E. coli* BL21 (DE3) cells. The culture was diluted 1 : 100 into fresh LB/kanamycin solution and grown to an OD_600_ of 0.6. Expression was induced with the addition of 0.1 mM isopropyl-*β*-D-1-thiogalactopyranoside (IPTG) and grown at 30°C, 200 rpm. Harvested cells were resuspended in lysis buffer, 20 mM potassium phosphate, pH 7.5, 150 mM NaCl, 10 mM imidazole, 25 *μ*M PLP, and a protease inhibitor cocktail (Roche). Cells were lysed by sonication on ice (Soniprep 150, 15 cycles of 30 seconds on followed by 30 seconds off). The lysate was centrifuged for 25 minutes at 16,000 rpm. The resulting supernatant was incubated with preequilibrated Ni resin (Ni-NTA Superflow, Qiagen) for 1 hour at 4°C. The protein eluted with 300 mM imidazole and was further purified by gel filtration (S200 HR, GE Healthcare) using 20 mM potassium phosphate, pH 7.5, 150 mM NaCl, and 25 *μ*M PLP buffer.

#### 2.2.4. Spectroscopic and Kinetic Measurements of Bacterial SPTs

All UV-visible spectra were recorded on Cary 50 UV-visible spectrophotometer (Varian) and analysed using Cary WinUV software (Varian). Prior to all UV-visible spectroscopies and assays, SPT was dialysed against fresh buffers containing 250 *μ*M PLP to ensure that the enzyme was in the PLP bound, holoform. Excess PLP was removed on a PD-10 (Sephadex G-25M) desalting column (GE Healthcare). For UV-visible assays the concentration of enzyme was 40 *μ*M, and the spectrophotometer was blanked with 20 mM potassium phosphate (pH 7.5) containing 150 mM NaCl at 25°C. Quartz cuvettes from NSG Precision Cells, Type 18-BM, with a light path of 10 mm and a sample volume of 500 *μ*L were used, and the spectra were collected from 800 nm to 200 nm. The release of CoASH from C16-CoASH during KDS formation was used to measure SPT activity as previously described [10]. Assays were performed on a 200 *μ*L scale on a 96-well format in a Biotek Synergy HT plate reader. Kinetic constants were calculated using Michaelis-Menten kinetics and using GraphPad Prism 6 software.

#### 2.2.5. Bacterial SPT Enzyme Activity by Measuring KDS Formation

SPT activity was also monitored by incorporation of [^14^C] L-serine into [^14^C]-KDS by a modification of a published method [10]. A final enzyme concentration of 25 *μ*M SPT (in 20 mM potassium phosphate buffer, pH 7.5, and 150 mM NaCl) was incubated with 20 mM U-^14^C L-serine (7400 Bq, 0.2 *μ*Ci, PerkinElmer) and 250 *μ*M palmitoyl-CoA in a final volume of 1 mL. The reaction was incubated at 37°C for 20 minutes, and then the reaction was quenched by the addition of NH_4_OH (final concentration 0.4 M). This was then extracted with an equal volume of CHCl_3_ : CH_3_OH (2 : 1, v : v). The sample was centrifuged at 13,000 rpm for 5 minutes, and the aqueous phase was discarded. The organic phase was left to evaporate at 50°C. The resulting lipid residue was resuspended in 15 *μ*L of CHCl_3_ : CH_3_OH (2 : 1, v : v) and spotted onto a Silica Gel 60 F_254_ TLC plate. Separation was carried out with a mobile phase of CHCl_3_ : CH_3_OH (2 : 1, v : v). The TLC plate was scanned with an AR-2000 imaging scanner and [^14^C]-labelled products quantified using the Laura 4 software (LabLogic).

#### 2.2.6. Determination of Dissociation Constants, Substrate and Product Quinonoid Detection, and SPT Activity Assays

Binding assays typically contained 40 *μ*M enzyme in 20 mM potassium phosphate (pH 7.5). Varying amounts of L-serine (0–40 mM) were added, and after addition of the substrate, the reaction mixture was allowed to equilibrate for 15 min at 25°C. The *K*_*d*_ values were calculated from plots of Δ425 versus L-serine concentrations by fitting to a hyperbolic saturation curve using Sigma Plot software

(1) $$\begin{array}{c} \Delta A_{\text{obs}}=\frac{\Delta A_{\max}\left[\text{serine}\right]}{K_{d}+\left[\text{serine}\right]}, \end{array}$$

where Δ*A*_obs_ represents the observed change in absorbance at 425 nm, Δ*A*_max⁡_ is the maximal absorbance change, [serine] represents L-serine concentration, and the *K*_*d*_ is the dissociation constant. Also, the formation of the quinonoid intermediate was followed. In this case, 50 mM L-serine and 1.5 mM S-(2-oxoheptadecyl)-CoA were mixed to the enzyme (40**** *μ*M) [10, 32], and the reactants were allowed to equilibrate for 15 min at 25°C (data not shown).

## 3. Results

### 3.1. Enzymatic Activity of Wild Type and HSAN1 hLCB2a Mutant SPT

**Figure 3:**
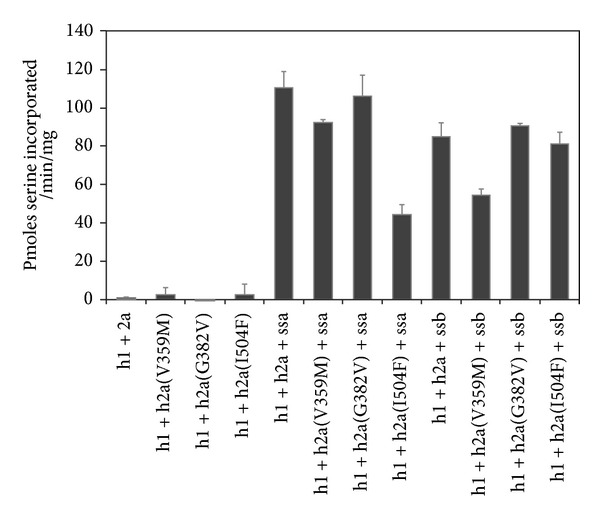
SPT activity in yeast microsomes expressing hLCB1 (h1) + hLCB2a (h2a, wt, or HSAN1 mutants) in the presence or absence of the small subunits ssSPTa (ssa) or ssSPTb (ssb).

In the report of the hLCB2a-causing HSAN1 mutants (V359M, G382V, and I504F), Rotthier et al. expressed mutant hLCB2a subunits in human embryonic kidney (HEK 293) cells and measured microsomal SPT activity [39]. They noted that each mutation reduced enzyme activity (above background) by different amounts (~40–100% activity) compared with wild type. In 2009, Han and colleagues discovered small subunits of SPT (ssSPTa and ssSPTb) that when coexpressed with hLCB1 and hLCBa increased human SPT activity by 50–100-fold [18]. These small subunit activators appear to play the same role as the small yeast protein Tsc3p also discovered by Gable and colleagues nearly ten years earlier [23]. To investigate the effect of the small subunits on the new HSAN1-associated hLCB2a mutants, we expressed each of the human mutant genes in yeast cells which had had the endogenous subunits of SPT knocked out. This strain, which completely lacks SPT activity, is not viable unless supplemented with a long chain base or a competent, active SPT complex. The yeast microsomes expressing wild-type hLCB1/hLCB2a in the absence of either small subunit displayed very low activity (<5 pmoles serine incorporated/min/mg protein) as predicted (Figure 3). Similarly, coexpression of each of the three HSAN1 hLCB2a mutants (V359M, G382V, and I504F) along with hLCB1 resulted in low activity, with G382V barely detectable above background mirroring the results reported by Rotthier and colleagues. Their activity values are in CPM (^14^C)/*μ*g protein so they are difficult to be compared with ours in absolute terms. When we coexpressed wild-type hLCB1 and hLCB2a in the presence of either ssSPTa or ssSPTb, the activity was indeed increased by 50–100-fold. Heterodimers containing the V359M and G382V mutant hLCB2a subunits were similarly activated by ssSPTa. In contrast heterodimers containing the I504 mutant hLCB2a subunit were activated to a lesser extent. When ssSPTb was expressed, heterodimers containing the G382V and I504F mutant hLCB2a subunits were activated to the same extent as wild-type heterodimers, but heterodimers containing the V359M mutant subunit were less well activated. Our interpretation of these data is that each of the new hLCB2a mutants had a small impact on SPT activity and that each mutant enzyme is still activated by the presence of the activating small subunits. The small impact of these new mutants is in contrast to the C133W, C133Y, and V144D mutations in hLCB1 but is similar to what was observed in the recently reported S331F mutation in hLCB1 [44].

### 3.2. Expression and Purification of HSAN1 hLCB2a Mutant Mimics

The three hLCB2a mutations that cause HSAN1 were individually introduced into the *Sp *SPT gene sequence (Figure 2), and the three mutant proteins were expressed using established protocols [10]. Two of the three mutants (*Sp *SPT V246M and G385F) were soluble. In contrast, the G268V mutant was found to be insoluble, and several attempts were made to improve the solubility of the protein (such as changing expression cell lines, induction at lower temperature, and different IPTG concentrations (data not shown)); however the majority of the protein remained in the cell pellet suggesting that this particular HSAN1 hLCB2a mutation leads to substantial protein misfolding of the bacterial mimic. The other two mutants (*Sp *SPT V246M and G385F) were obtained in milligram quantities and purified via nickel affinity chromatography as previously described for the wild-type SPT [10]. Both V246M and G385F behaved as dimers by size exclusion chromatography (molecular weights ~90 kDa) and displayed elution profiles similar to wild-type SPT (data not shown). 

### 3.3. Spectroscopic Properties of WT SPT and HSAN1 Mutant Mimics

**Figure 4:**
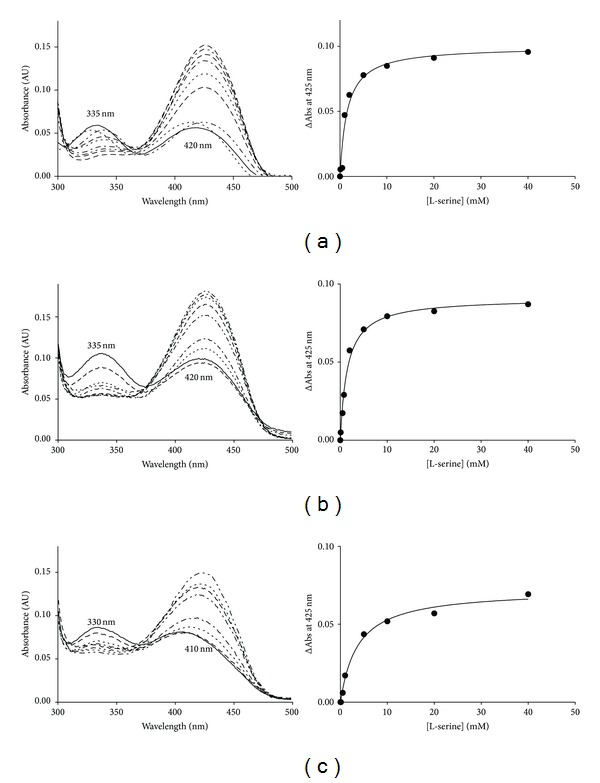
UV-visible analysis of *Sp* SPT wild-type and mutant mimics. Absorbance spectra of (a) *Sp *SPT wild type, (b) SPT V246M, and (c) SPT G385F. In the wild-type spectrum (a), the enolimine (335 nm) and ketoenamine (420 nm) forms of the external aldimine are shown. The solid line in each spectrum is the holoform of the enzyme (40 *μ*M SPT, 20 mM potassium phosphate buffer (pH 7.5), and 150 mM NaCl, 25°C). Increasing concentrations of L-serine were added (0, 0.5, 1, 2, 5, 10, 20, and 40 mM; dotted and dashed lines), and the spectrum was recorded after 15 minutes.

Formation and breakdown of many of the intermediates within the SPT mechanism (Figure 1) can be monitored by UV-visible spectroscopy due to the yellow colour of the PLP-bound enzyme. Initial analysis of the *Sp *SPT V246M (Figure 4(b)) and G385F (Figure 4(c)) by UV-visible spectroscopy revealed that both HSAN1 mutants display spectra similar to wild-type SPT, with two absorbance maxima at around 335 and 420 nm (Figure 4(a)). These peaks are characteristic of the holo-, PLP-bound internal aldimine form of the enzyme, arising from the two tautomeric forms (enolimine and ketoenamine resp.) of PLP. These results confirm that both mutants were purified with the PLP cofactor covalently bound in the enzyme active site as an imine to Lys265. However, on closer inspection it is clear that there are subtle differences in the spectral profile of SPT G385F compared with the wild-type enzyme. In contrast to the wild-type SPT, the UV-visible spectrum of SPT G385F shows a significant blue shift with maximum absorbance values of 330 nm and 410 nm (Figure 4(c)). This suggests that mutation at position 385 has had an impact on the way the PLP cofactor binds at the active site. 

The internal aldimine/PLP-bound form of SPT can be displaced with increasing concentrations of the substrate L-serine to form a SPT:PLP-L-serine external aldimine which can be easily monitored by observing the change in absorbance at 425 nm. Analysis of the wild-type SPT and both mutants led to the determination of the apparent L-serine dissociation constants (*K*_*d*_^ser^, Table 2). Binding studies were also carried out with L-alanine and glycine (data not shown). At physiological concentrations, L-serine was able to form the external aldimine in wild type and both mutant forms of SPT. Wild-type SPT binds L-serine with a *K*_*d*_^ser^ of 1.1 mM (as obtained previously, Raman et al. [10]), and a similar *K*_*d*_^ser^ value of 1.5 mM was obtained for the V246M mutant. This analysis supports the initial observation (based on UV-visible data) that PLP is bound in the same environment as that of the wild-type enzyme. In contrast, a *K*_*d*_^ser^ of 4.7 mM was obtained for mutant G385F, a ~4-fold increase in comparison to the wild-type SPT, suggesting that this mutation has had some impact on both PLP and substrate binding. We have shown previously that the wild-type bacterial SPT is unable to use glycine or L-alanine as substrates to allow formation of deoxy-SLs to appreciable levels, and neither the V246M nor G385F mutant binds to L-alanine or glycine (data not shown). 

### 3.4. Kinetic Analysis and KDS Formation of SPT WT and HSAN1 Mutant Mimics

In order to ascertain the effect that each of the HSAN1 hLCB2a mutant mimics has on enzyme catalysis, Michaelis-Menten kinetic analyses were performed for the wild-type V246M and G385F enzymes using a DTNB assay that monitors formation of the CoASH product (Figure 1). The wild-type SPT bound L-serine and palmitoyl-CoA (C16-CoA) with *K*_*m*_ values of 1.6 mM and 35.6 *μ*M, respectively (Table 2). The enzyme turned over with a *k*_cat_ of 1.14 s^−1^ and efficiency (*k*_cat_/*K*_*m*_) of 712 M^−1^s^−1^ for L-serine and 32022 M^−1^s^−1^ for the acyl-CoA—all in good agreement with published values [10]. In contrast, we measured lower *k*_cat_/*K*_*m*_ values of 176 and 136 M^−1^s^−1^ for L-serine (4- and 5-fold) and 3437 and 7854M^−1^s^−1^ for C16-CoA (9- and 4-fold lower) and for the V246M and G385F mutants, respectively (Table 2). In addition, we monitored product KDS formation directly by using incorporation of ^14^C L-serine. Both mutants were less active and displayed 84% (V246M mutant) and 15% (G385F) KDS production relative to the wild-type SPT enzyme.

### 3.5. Substrate Quinonoid Formation in SPT WT and HSAN1 Mutants

The ability of the wild-type enzyme and mutants to generate a key carbanion/quinonoid species was tested using the C16-CoA thioether analogue, S-(2-oxoheptadecyl)-CoA. Addition of the analogue to the PLP-L-serine external aldimine form of the wild-type SPT led to the appearance of a peak at ~495 nm which is thought to be the substrate quinonoid [45]. Under the same conditions, both V246M and G385F generate a quinonoid peak confirming that these mutants can catalyse formation of this key intermediate (data not shown).

### 3.6. Modelling the Structural Impact of HSAN1 Mutations

**Figure 5:**
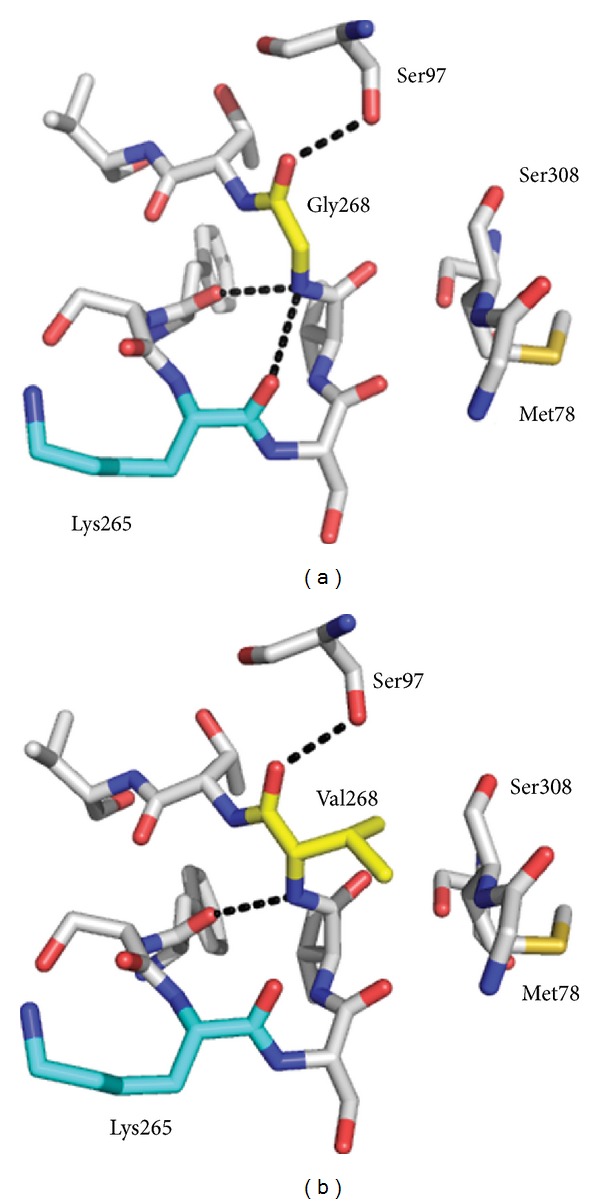
Structural models of the HSAN1 mutant mimic *Sp *SPT G268V using the *Sp *SPT PLP-L-serine external aldimine structure (PDB:2W8J). (a) Wild-type *Sp* SPT highlighting interactions within 5 Å of G268. (b) Mutated residue Val 268 showing new interactions within 5 Å. The models and figures were generated using PyMol software.

Unfortunately, neither of the SPT V246M nor G385F HSAN1 mutant mimics crystallised (under the same conditions as wild type [7, 10] or by screening for new conditions). Therefore, to rationalise the impact that each of the HSAN1 hLCB2a mutations has on the structures of these altered enzymes, modelling was carried out based upon the crystal structure of holo-SPT (PDB:2JG2) [7] and the SPT:PLP-L-serine external aldimine complex (PDB:2W8J) [10]. It is worth noting that the PLP-binding site is at the subunit interface between the two monomers, and the active site is formed by residues from both subunits. We used the “mutagenesis” tool within PyMol [46] to replace the wild-type side chain with each mutant side chain and applied the default conformational constraints to produce a structural model. We chose to highlight interactions that were within 5 Å of each mutant residue of interest. The amide backbone of G268 makes polar contacts with the side chain of S97 and the backbone of F263 and most importantly the backbone of the crucial K265 residue involved in PLP binding (Figure 5). The model predicts that these polar contacts to S97 and F263 are maintained after inclusion of the isopropyl side chain of the G268V mutant; however, it loses the polar contact with the K265 residue. In addition to this, several new interactions are predicted to occur within 5 Å of the V268 mutant side chain in comparison to G268. The most interesting/important of these is the additional contact made with the backbone of M78 (from the same subunit) that leads to a severe steric clash with the side chain of V268. This interaction is not present in the wild-type structure and suggests that the bulky hydrophobic valine residue cannot be accommodated at this position. This structural change may well be the cause of the observed detrimental effect on protein solubility.

**Figure 6:**
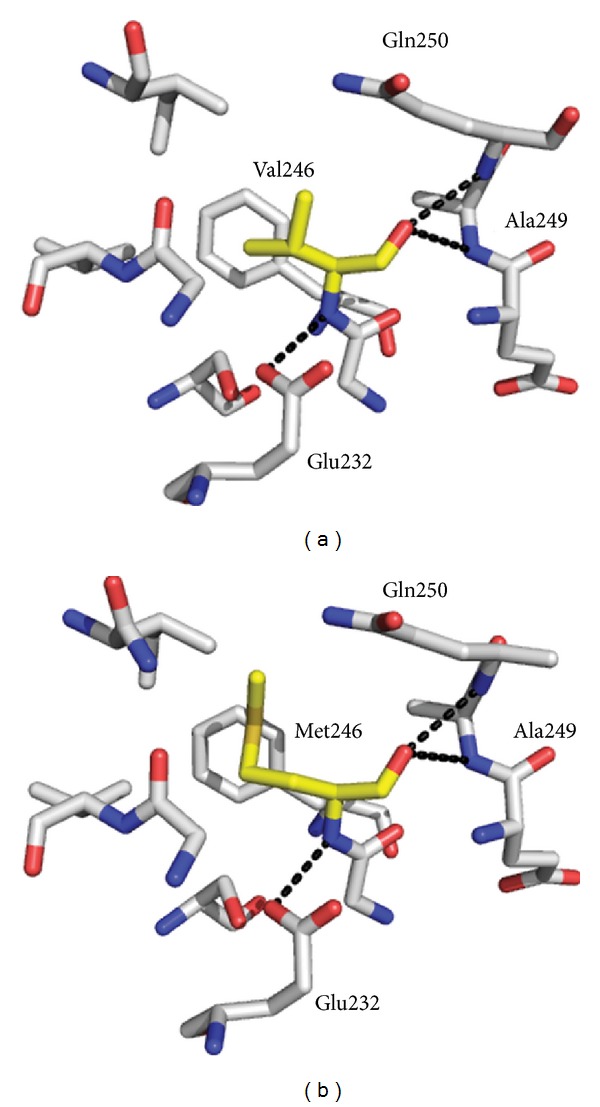
Structural models of HSAN1 mutant mimic *Sp* SPT V246M mutation using the *Sp *SPT-L-serine external aldimine structure (PDB:2W8J). (a) Wild-type V246. (b) Mutation of residue 246 to a methionine. Residue 246 is shown in yellow. The models and figures were generated using PyMol software.

Position G385 is shown to be on the surface of SPT and solvent exposed (Figure 6). Mutation of this residue to F385 may result in this hydrophobic side chain preferring a buried hydrophobic pocket. G385 resides in an important, conserved SPT motif 379-PPATPAGTFLLR-390 that is known to undergo conformational change during the catalytic cycle. Residue R378 is known to bind the carboxylate of the L-serine substrate in the PLP-L-serine external aldimine complex and to do so undergoes large movement brought about by the structural changes of the PPATP loop [10]. Mutation of R378 impacts both substrate binding and catalysis; our model suggests that the introduction of F385 would alter the conformational flexibility of the enzyme. This is indeed the case since we observed a ~5-fold decrease in efficiency (*k*_cat_/*K*_*m*_^L-ser^) of the G385F mutant compared with wild type (Table 2).

**Figure 7:**
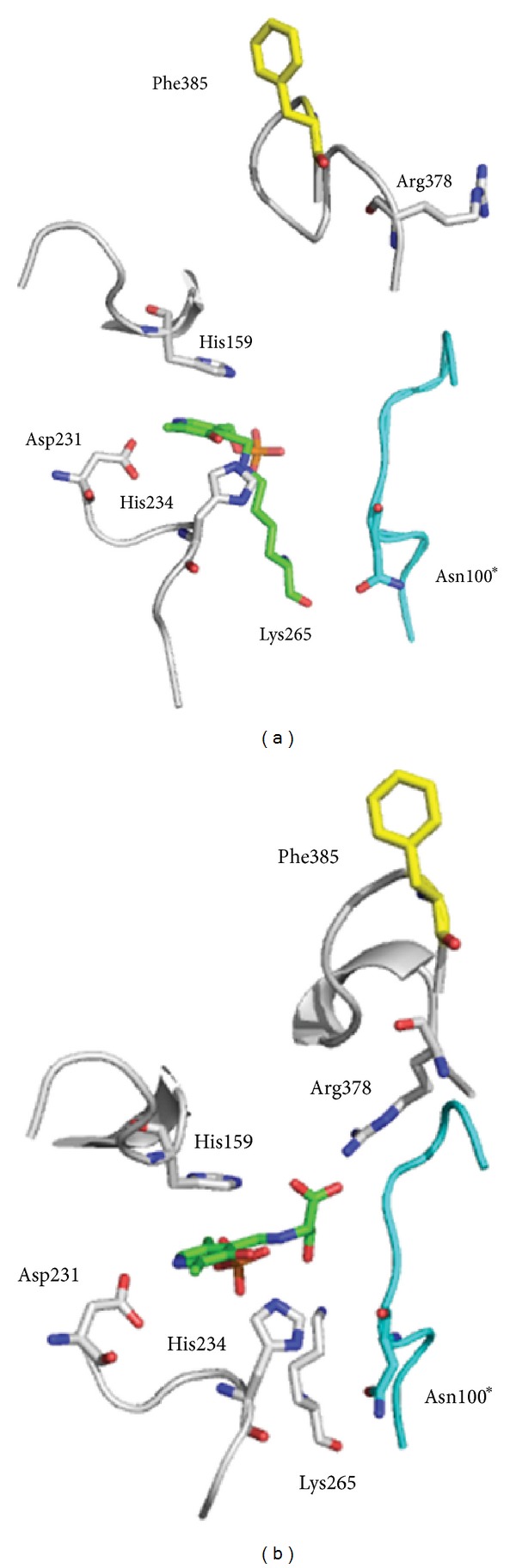
Structural models of the *Sp* SPT G385F mutant mimic. (a) *Sp *SPT holostructure (PDB:2JG2) and the L-serine external aldimine structure (PDB:2W8J). Residues from monomer one are shown in white, residues from monomer two are shown in teal, the PLP cofactor is shown in green, and the mutated glycine to phenylalanine residue is shown in yellow. The models and figures were generated using PyMol software.

The side chain of residue V246 is in a hydrophobic pocket in close proximity to F239 and V204, and the amide backbone makes polar contacts with the side chain of E232 and the backbone amides of A249 and Q250 (Figure 7). In the model, mutation of this residue to a methionine does not cause any severe alterations or clashes, and the M246 side chain is accommodated in the same pocket. Nevertheless, this mutation does have some impact on the palmitoyl-CoA binding by reducing the  *K*_*m*_  by ~4-fold and (*k*_cat_/*K*_*m*_^PCoA^) approximately by 9-fold.

## 4. Discussion

HSANs are a rare group of disorders of the peripheral nervous system (PNS) [47]. They arise through a series of diverse genetic mutations and present a broad range of clinical symptoms. Overall, HSANs lead to neurodegeneration of the PNS, and the current working hypothesis is that HSAN1 is caused by mutations in subunits of SPT that impact the structure and function of the enzyme [29–31, 34, 48]. An appreciation of the complexity of the eukaryotic SPT enzyme has increased over the past 4-5 years with the identification of new regulatory subunits (such as the small subunits ssSPTa and ssSPTb and the ORMs), but at its heart it requires the homologous subunits hLCB1 and hLCB2a which come together to form a heterodimeric core [24–26]. It is thought that, since hLCB2 contains residues that are conserved amongst other PLP-binding enzymes, it is essential for the covalent attachment of PLP and SPT catalyses, whereas hLCB1, which lacks the conserved residues, plays important roles in catalysis and regulation [6]. Mutations that knock out SPT activity all together are lethal emphasizing the importance of *de novo* SL biosynthesis. However, linkage analysis of patients/families with HSAN1 led to the identification of disease-causing mutations in the hLCB1 subunits of SPT [29, 30]. More recently additional disease-causing mutations have been found in hLCB1 [33, 36–38] as well as in the hLCB2a subunit [39]. Importantly, it was discovered that, while mutations such as C133W and C133Y in hLCB1 greatly-reduced SPT activity, the pathophysiology of HSAN1 is most probably due to a gain of function that results in the ability to utilize alanine and/or glycine as substrates [29, 30]. Patients with the hLCB1 C133W/C133Y mutations generate deoxy-SLs (1-deoxy and 1-deoxymethyl) that are present in high concentrations compared to normal individuals. This was subsequently confirmed in mouse models of the disease [32, 49]. It is thought that these neurotoxic deoxy-SLs are built up over time to such concentrations that they lead to nerve damage and breakdown. Since these deoxy-SLs cannot be phosphorylated (to sphingosine 1-phosphate (S1P)), they cannot be degraded by the S1P lyase and accumulate in cells. The thinking is that different HSAN1 mutant SPTs (where either the hLCB1 or hLCB2a proteins are altered) have differing impacts on the activity with L-serine as a substrate, but where there is a decrease, it is still above the threshold required for cell survival. More importantly, these mutations have differing influences on the promiscuity of the mutant enzyme such that it can also accept other amino acid substrates. A discussion of this interesting model by SPT experts at a symposium organised by a family afflicted by the disease (Deater Symposium, Boston, April 2008) also suggested that a potentially simple HSAN1 “cure (treatment)” would be increasing dietary serine thereby increasing the intracellular pool of L-serine to increase the ratio of serine/alanine thereby reducing the synthesis of the toxic deoxy-SLs. Indeed, this appears to be the case with the recent success of early clinical trials of oral L-serine supplementation that reduced deoxy-SL levels in serum and also led to an improvement in motor and sensory performance in mice [50].

 Rotthier and colleagues presented data that the new HSAN1 hLCB2a mutants (V359M, G382V, and I504F) reduce SPT activity when expressed in HEK293 cells. They also measured the deoxy-SL levels in HEK293 cells and the lymphoblasts derived from HSAN1 patients carrying the G382V and I504F mutations. In both cases deoxy-SL levels were very high in agreement with the toxic SL hypothesis. We also wanted to measure the impact of the small subunits on the HSAN1 hLCB2a mutants. As well as activating basal SPT activity, the small subunits modulate the acyl-CoA chain-length specificity of the SPT [18]. We found that coexpression of either ssSPTa or ssSPTb increased basal activity of wild-type heterodimers and those containing all three hLCB2a mutants to varying degrees. In our recent paper we studied the kinetics of SPT containing the hLCB1^C133W^ HSAN1 mutant subunit and found that it had similar affinity for L-serine as did wild-type SPT [31]. Surprisingly, the same was true for L-alanine indicating that the major impact of the C133W mutation is to enhance activation of the amino acid substrate for condensation with the acyl-CoA substrate. A similar kinetic analysis has not yet been done for the hLCB2a mutants. We do not discuss the ability of these new HSAN1 mutants to use glycine and/or L-alanine as substrates here as it requires a more detailed kinetic analysis of nine variables; the four subunits (SPT1/2 and ssSPTa/b) with five substrates (L-serine, glycine, L-alanine, and C14 and C16 acyl-CoAs), are the subject of current work. However, we felt that valuable insights would be gained by using the soluble bacterial SPT as a model to make mutant mimics. We previously studied the hLCB1 C133W mutant by mapping this residue to N100 of the *S. paucimobilis* SPT [10]. The side chain of N100 is close to but not in the active site of SPT, which lies at the subunit interface of the soluble bacterial homodimer. Nevertheless, it does contact the amide backbone of the key, conserved active site lysine (K265) that binds the PLP as an internal aldimine, and is also thought to act as the base that removes the proton from C-*α* of L-serine (Figure 1). Replacement of N100 with either the bulky tryptophan or tyrosine residue found in C133W and C133Y might be expected to be disruptive to the structure and activity of the homodimeric enzyme. Indeed, when we made the N100W and N100Y mutants, we noted that PLP binding was altered, and both the binding constants (*k*_*m*_) and catalytic activities (*k*_cat_) were lowered significantly compared to the wild type. Moreover, we determined the crystal structure of the N100Y mutant in its PLP-L-serine external aldimine form, and a number of structural changes were observed; a key residue (Arg378) that binds the L-serine carboxylate was in the “swung out” conformation, the important PPATP loop was in a twisted conformation, and the side chain of the mutant mimic Y100 residue was now impacting the opposite subunit. This study revealed that mutations in one subunit can impact the overall structure and activity of the dimer and provided us with some insights as to how HSAN1 causing mutations may affect the human hLCB1/hLCB2a complex [10].

 Two of the three new hLCB2a HSAN1 causing mutations (V359M and G382V) are found at residues that are strictly conserved in LCB2s from human, mouse, fly, rat, zebra fish, yeast, and bacterial SPT [39]. The third, I504, is less highly conserved; in fly (*Drosophila melanogaster*) it is a methionine, and in bacterial *Sp *SPT enzyme it is a glycine. The HSAN1 causing mutations in hLCB2a (V359M, G382V, and I504F) correspond to bacterial *Sp *SPT mutationsV246M, G268V and G385F, respectively (Figure 2). G268 is three residues away from the essential active site residue K265 and in the 3D structure makes contact with the backbone of this residue. It was not surprising that introduction of G268V resulted in a drastic reduction in protein solubility, presumably due to misfolding during recombinant expression. Modelling studies reveal that this residue is also near to the side chain of residue N100 from the opposite subunit of the homodimer that we discussed above (Figure 5). In mutating G268 to valine, polar contacts to the K265 residue are lost. A plausible explanation for the misfolding of the G268V mutant is that it disrupts the polar contacts with the key lysine residue. The global fold of the protein could also be adversely affected by an apparent steric clash between the side chain of valine in position 268 and the backbone of Met78 (Figure 5(b)). In the initial report of the holo-*Sp* SPT structure by Yard et al., active site residues were identified, and G268 was also identified as an essential active site residue which hydrogen bonds with the backbone oxygen of K265 [7]. The G268V model predicts that this H-bond is lost and is therefore not surprising that this mutation has such a detrimental impact on the bacterial enzyme (Figure 5(b)).

 In contrast to the G268V mutant, modelling studies do not give us clear insight as to the structural basis for the HSAN1 phenotype in the V246M or G385F mutants. These mutations appear to be more subtle. However, analysis of the PLP cofactor binding and enzyme kinetics of these two mutant mimics does provide some clue as to why these changes would cause alterations of the hLCB2a subunit that would lead to the HSAN1 phenotype. For the V246M mutant, structural models show that the V246 residue is far from the PLP active site, and polar contacts with E232, A249, and Q250 are retained after mutating the residue to a methionine (Figure 6). The UV-visible spectrum of this mutant is very similar to wild-type SPT and suggests that the PLP is bound in a similar orientation to the wild-type enzyme (Figure 4). The apparent *K*_*d*_^L-ser^ is unchanged by the incorporation of the HSAN1 mutation, suggesting that, in this case, the key L-serine external aldimine intermediate is formed in the same way as in the native SPT (Figure 1). However, this mutation does have a detrimental effect on the observed catalytic rate and efficiency, which again are 3-fold and 9-fold lower than wild-type enzyme (Table 2). This V246M mutant mimic also displays a significantly higher *K*_*m*_ for the acyl-CoA thioester substrate, suggesting that binding of this substrate is compromised at least subtly in this mutant. This is useful information about a residue potentially involved in binding the second substrate since, to date, we still do not have a structure of the *Sp* SPT PLP complex with palmitoyl-CoA bound.

In the structure of *Sp* SPT, residue G385 is surface-exposed (Figure 7). For the G385F mutant it is plausible that replacing glycine with the large, hydrophobic phenylalanine residue may lead it to adopt an alternative conformation. This has the potential to substantially affect the architecture of the surrounding structure, such as the important PPATP loop which undergoes large conformational changes during the catalytic mechanism. However, without crystallographic data for this mutant, it is impossible to confidently propose any effect of a potential realignment at this position. In the case of G385F, UV-visible analysis suggests that PLP binding is altered and that the cofactor is in a different environment compared to the wild-type enzyme. Furthermore, the apparent *K*_*d*_^L-ser^ was ~5 times higher for this mutant indicating that serine binding is affected by this mutation (Table 2). Both the catalytic rate and catalytic efficiency are also significantly reduced (3-fold and 4-fold, resp.) demonstrating that catalysis is also impaired.

## 5. Conclusion

Our results have shed light on how the three recently-discovered HSAN1 causing hLCB2a mutants affect the SPT complex in the presence of the activating small subunits (ssSPTa and ssSPTb). To explore the impact of these mutations on PLP binding, SPT structure, and activity, we also use mutants of the soluble homodimeric bacterial SPT as human SPT mimics to reveal that each mutant has a different impact on the enzyme. These range from subtle alterations to how PLP sits within the SPT active site, lowering substrate binding and catalytic activity through to a complete loss of solubility all brought about by a single change. New SPT mutations that result in HSAN1 are being discovered periodically; for example, another on the hLCB2a subunit, A182P, was recently characterised that had reduced SPT activity, a higher preference for L-alanine, and increased plasma deoxy-SLs levels [42]. Exactly how these HSAN1 mutations cause the human hLCB1/hLCB2a/ssSPT complex to lose its exquisite specificity for L-serine and also accept L-alanine and glycine to form deoxy-SLs will require in-depth structural and kinetic analyses of the purified wild-type and mutant SPT complexes.

## Figures and Tables

**Table 1:** Overview of currently known hLCB1 and hLCB2a HSAN1 related mutations.

Mutant	*Sp* SPT residue	Location in *Sp *SPT	Clinical features	Reference
hSPT1				
C133Y	N100	Active site	Sensory neuropathy, ulceromutilations, and lancinating pains.	[29, 30]
C133W	N100	Active site	Sensory neuropathy, ulceromutilations, and lancinating pains.	[29, 30]
C133R	N100	Active site	Sensory neuropathy.	[36]
V144D	D121	Surface exposed	Sensory neuropathy, ulceromutilations, and lancinating pains.	[30]
S331F	H278	Surface exposed	Ulcerations, mental retardation, hypotonia, severe growth and mental retardation, vocal cord paralysis, and gastroesophageal reflux.	[33, 37]
S331Y	H278	Surface exposed	As S331F.	[38]
A352V	S308	Not surface exposed	Sensory neuropathy, ulceromutilations, and lancinating pains.	[33, 37]
G387A	E343	Surface exposed	No disease associated.	[40, 41]

hSPT2				
V359M	V246	Not surface exposed	Sensory neuropathy, ulceromutilations.	[39]
G382V	G268	Active Site	Sensory neuropathy, ulceromutilations.	[39]
I504F	G385	Surface exposed	Sensory neuropathy, ulcerations, osteomyelitis, and anhidrosis.	[39]
A182P	T79	Not surface exposed	Sensory neuropathy.	[42]

**Table 2:** Kinetic parameters of wild-type *Sp* SPT and mutants.

Enzyme	k_cat_ × 10^−2^ (s^−1^)	K_m_^L-ser^ (mM)	K_*m*_^PCoA^ (μM)	k_cat_/K_m_^L-ser^ (M^−1^ s^−1^)	k_cat_/K_*m*_^PCoA^ (M^−1^ s^−1^)	K_*d*_^L-ser^ (mM)
SPT WT	114 ± 2.0	1.6 ± 0.09	35.6 ± 2.0	712	32022	1.1 ± 0.1
SPT V246M	44 ± 1.2	2.5 ± 0.2	128.0 ± 10.0	176	3437	1.5 ± 0.1
SPT G385F	41 ± 2.0	3.0 ± 0.5	52.2 ± 8.0	136	7854	4.7 ± 0.1
SPT G268V	—	—	—	—	—	—

## Abbreviations

SPT: Serine palmitoyltransferase
PLP: Pyridoxal 5′-phosphate
SL: Sphingolipid
LCB: Long chain base
*Sp:*: * Sphingomonas paucimobilis*
HSAN1: Hereditary sensory and autonomic neuropathy type I
ss: Small subunit
AU: Absorbance unit.
